# 

**Published:** 1895-06-29

**Authors:** 


					JUNE iiyTH, isao.
^ DDinr TlifOnPklrvt W W ^ WITH EXTRA NURSING SUPPLEMENT
PRICE TWOPENCE BUI P? un 10..?...???<*...
NM57;v;rxVlil,-Tu'ne~29Uri895.^i; JOL JPJ
i"Urfrt Rt the GfiBfiral PoBt Offlca s? % NHvhV>-ier. Ditto par Annum, 8s. 6d.,post ftrea.
)rmch hospital
WITH EXTRA NURSING SUPPLEMENT. 29?, 1895.

				

